# Taxonomic study on two Chinese species of the genus *Cerynia* (Hemiptera, Fulgoromorpha, Flatidae)

**DOI:** 10.3897/zookeys.431.6987

**Published:** 2014-08-06

**Authors:** Weicheng Yang, Xiangsheng Chen

**Affiliations:** 1 Institute of Entomology, Guizhou University; Special Key Labortory for Development and Utilization of Insect Resources of Guizhou,Guiyang,P.R.China,550025; 2 School of Life Sciences, Guizhou Normal University, Guiyang, Guizhou, P. R. China, 550001; 3 College of Animal Sciences, Guizhou University, Guiyang, Guizhou, P.R. China,550025

**Keywords:** Flatidae, planthopper, morphology, distribution, China

## Abstract

Male genitalia of the species *Cerynia lineola* Melichar is described and illustrated for the first time from China. Colour polymorphism in a planthopper species *Cerynia maria* (White, 1846) is investigated based on some specimens from China, and a map showing the geographic distribution of this species is also provided. The examined specimens are deposited in the Institute of Entomology, Guizhou University, Guiyang, China (GUGC).

## Introduction

The flatid genus *Cerynia* (Hemiptera: Fulgoromorpha: Flatidae) was established by Stål (1862) with *Flata albata* Stål, 1854 from Malaya as its type species. Subsequently, Distant (1879) proposed the new combination *Cerynia maria* (White, 1846). Melichar (1901) described three new species: *Cerynia fulgida* from Indonesia, *Cerynia trilineata* from Indonesia and *Cerynia lineola* from China, and proposed the new combination *Cerynia monacha* (Gerstaecker, 1895). Schmidt (1904) described a new species *Cerynia nigropustulata* from Indonesia. China (1925) described a new species *Cerynia parnassioides* from China. Ossiannilsson (1940) described a new species *Cerynia bilineata* from Java. Medler (1996) added a new species *Cerynia mixana* based on the Walker’s (1851) identification specimen, and later he (1999) placed *Cerynia nigropustulata* Schmidt, 1904 as a junior synonym of *Cerynia maria* (White, 1846). Recently, Wang and Peng (2007) described a new species *Cerynia digitula* from China. Until now, 10 species were known and all species are distributed in the Oriental Region, including 4 from China and most are distributed in southern China. Among these species, the male genitalia of *Cerynia lineola* has not been described and illustrated.

Here, we described and illustrated the male genitalia of *Cerynia lineola* Melichar, 1901 based on specimens from China (Hainan) for the first time, and provided data on colour polymorphism of the species *Cerynia maria* (White, 1846) in populations from China. The examined specimens are deposited in the Institute of Entomology, Guizhou University, Guiyang, China (GUGC).

## Material and methods

Specimens were collected by sweeping net. Dry specimens were used for the description and illustration. External morphology was observed under a stereoscopic microscope and characters were measured with an ocular micrometer. Color pictures for adult habitus were obtained by KEYENCE VHX-1000 system. The genital segments of the examined specimens were macerated in 10% NaOH and drawn from preparations in glycerin jelly using a Leica MZ 12.5 stereomicroscope. Illustrations were scanned with Canon CanoScan LiDE 200 and imported into Adobe Photoshop CS3 for labeling and fig composition.

Terminology of morphological and genital characters follow Peng et al. (2012).

## Taxonomy

### Cerynia lineola

Taxon classificationAnimaliaHemipteraFlatidae

Melichar, 1901

Figs 1–6

Cerynia lineola Melichar, 1901: 221; Chou et al. 1985: 89; Wang and Peng 2007: 934.

#### Material examined.

**CHINA:** 1♂1♀, Hainan Prov., Jianfengling, 5 May 2013, coll. Jichun Xing and Jiankun Long.

#### Description.

Male body length 21.5mm. Female body length 24mm.

Head light brown, clypeus slightly dark, antenna dark brown, eyes reddish brown. Pronotum light brown, lateral areas with a big brown marking. Mesonotum slight yellow to yellow, middle area with two small dark brown markings near anterior margin, lateral area with three big dark brown spots. Tegula dark brown. Forewings pink, veins yellow, with a transverse black stripe from apex of clavus to vein M, hindwings white. Legs black, hind tarsus dark brown.

Head including eyes slightly narrower than pronotum. Vertex depressed, nearly rectangle, with ratio of width at widest part to length in midlle line 8.4, median carina obsolete, anterior and posterior margins carinate; lateral margin together lateral margin of frons distinctly foliate. Ratio length of frons in middle line to width at widest part 1.3; base margin of frons broadly convex, apical margin nearly straight, lateral margin sinuate and distinctly foliate; disk of frons without middle carina. Clypeus subtriangular, with disk elevated in the middle, ratio length of frons in middle line to length of clypeus 1.5; face depressed at frontoclypeal suture. Pronotum with anterior margin broadly convex, middle carina obsolete, middle area of disk broad and explanate, lateral area gradually narrowing anteriorly and lateral margin nearly straight. Mesonotum with lateral carinae roundly curved, with ratio of length in middle line to length of pronotum and vertex combined 3.0. Veination of forewing complex, with dense crossveins, ratio length to width 1.8, ratio width to width of coStål cell 1.8, anterior margin slightly convex, apical margin broadly round, vein Sc with many branches, of which reaching anterior margin, R+M+Cu forking at base, apical cells distinctly long, clavus terminating at apical 2/5 of forewing. Surface of forewings with sparse granules, area of clavus much denser. Hind tibiae with two lateral spines and seven apical spines.

***Male genitalia*.** Pygofer with dorsal margin distinctly shorter than ventral margin in lateral view. Anal segment, about 90° bent down at basal 1/3, apical margin round in lateral view. Genital style slightly subrectangular with a short process at dorsal apex, ventral margin sinuate. Aedeagus in dorsal view symmetrical; phallobase tubular at base in lateral view, dorsolateral lobe slender, ventral lobe short; theca bearing one process at subapex on each side, apex each side with two processes, one of them longer than phallus.

#### Distribution.

China (Hainan, Yunnan); Japan; Vietnam.

#### Remarks.

This species differs from other species by the apex of theca with a pair of long and short processes in dorsal view, and each side also with a process at subapex.

#### Note.

The male genitalia of this species is described and illustrated for the first time.

**Figures 1–6.**
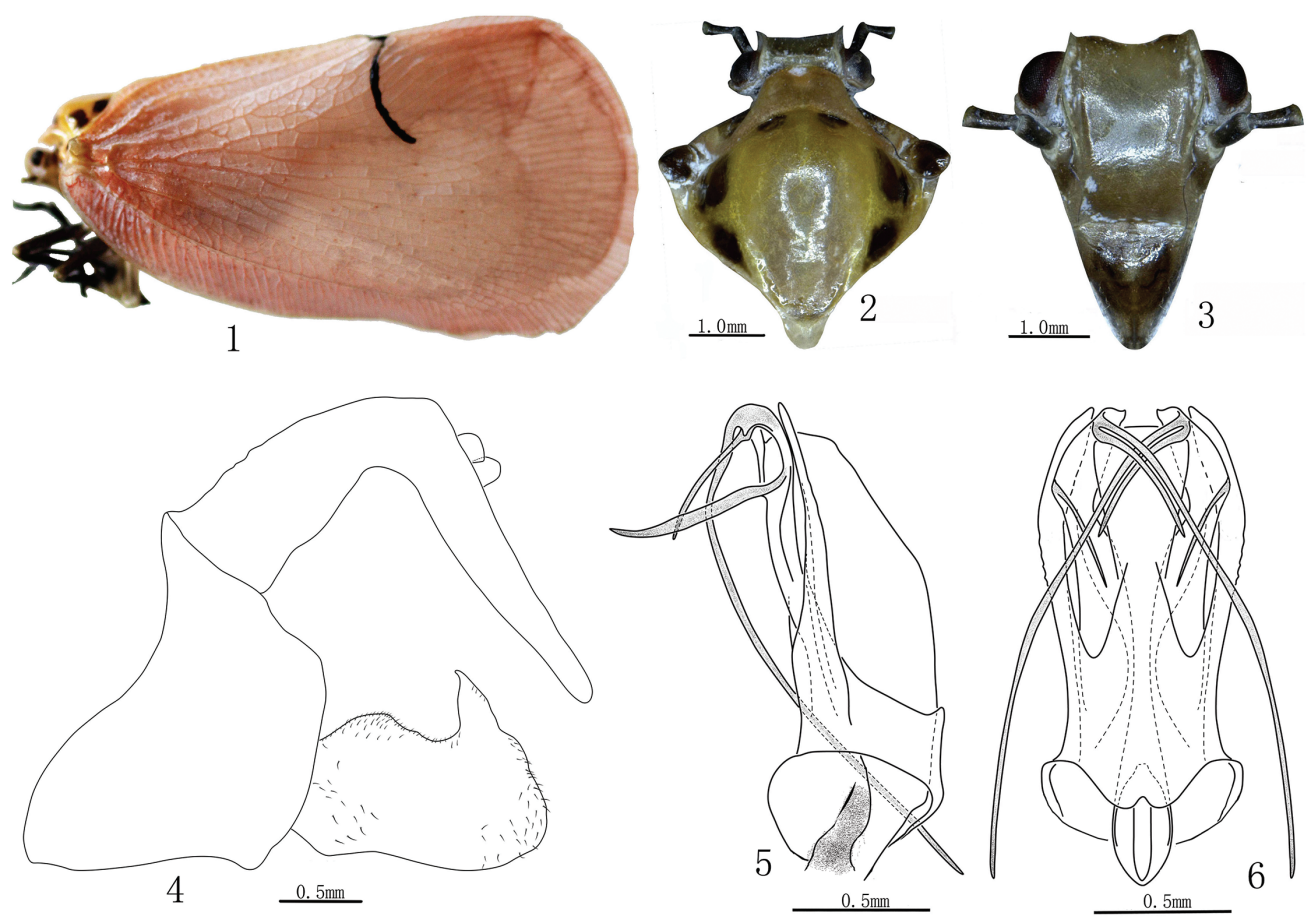
*Cerynia lineola* Melichar, 1901. **1** Adult habitus, lateral view **2** Head and thorax, dorsal view **3** Face **4** Pygofer, anal segment and genital style, lateral view **5** Aedeagus, lateral view **6** Aedeagus, dorsal view.

### Cerynia maria

Taxon classificationAnimaliaHemipteraFlatidae

(White, 1846)

Figs 7 – 9

Poeciloptera maria White, 1846: 25.
Flata maria Walker, 1851: 436.
Cerynia maria Distant, 1879: 38; Chou et al. 1985: 88; Wang and Peng, 2007: 934.
Cerynia maria var *completa* Medler, 1996: 20.
Cerynia nigropustulata Medler, 1999: 20.

#### Description.

Body length (incl. forewing):

A form: male: 15.2–16.5 mm(n=23); female: 17.0–18.5mm (n=10)

B form: male: 15.0–16.2 mm(n=7); female: 16.2–18.0mm. (n=8)

C form: male: 14.8–15.8 mm(n=3); female: 16.0–17.8mm. (n=12)

D form: male: 15.1 mm(n=1); female: 17.5mm. (n=1)

**Figure 7.**
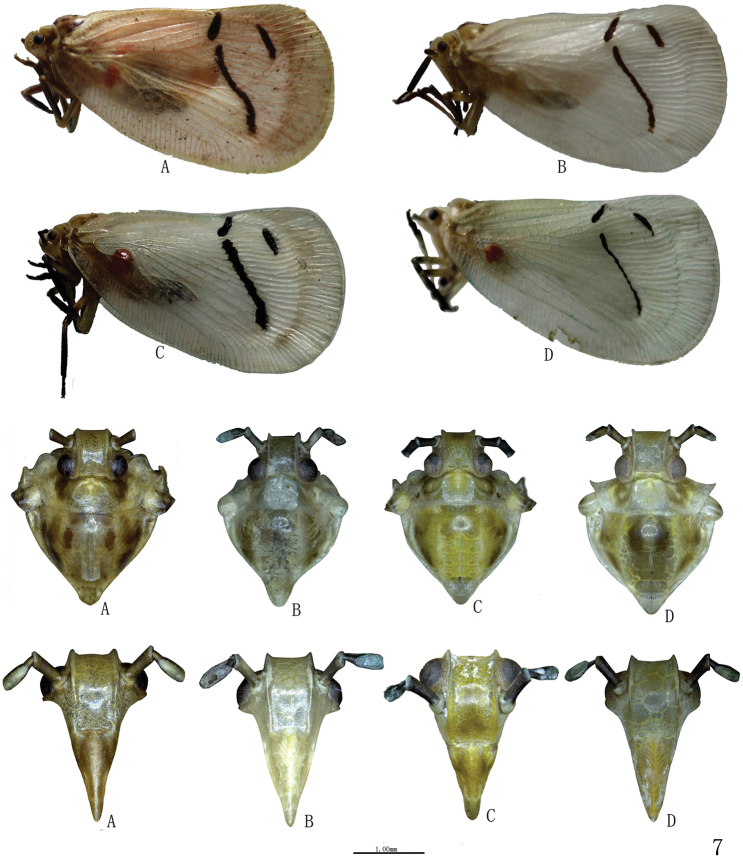
*Cerynia maria* (White, 1846) Colour pattern variations of adult habitus (lateral view), head and thorax (dorsal view), face (ventral view) **A** (Guizhou, Guiyang; Guizhou, Luodian; Guizhou, Yanhe, Lijiaba; Guizhou, Maolan, Banzhai; Guizhou, Daozhen, Xiannvdong; Guizhou, Ziyun, Getuhe) **B** (Guangxi, Damingshan; Guizhou, Yanhe, Maojiacun; Guizhou, Xingyi, Muka) **C** (Guangxi, Longzhou; Guangxi, Daxing, Encheng; Guizhou, Liping, Taipingshan) **D**(Yunnan, Menglun).

Head and thorax colour pattern yellow-brown, with variable black to dark brown markings on mesonotum (Fig. 7).

Head including eyes slightly narrower than pronotum. Vertex depressed, nearly rectangle, median carina obsolete, anterior and posterior margins carinate; lateral margin together lateral margin of frons distinctly foliate. Base margin of frons broadly convex, apical margin nearly straight, lateral margin sinuate and distinctly foliate; disk of frons without middle carina. Clypeus subtriangular, with disk elevated in the middle, face distingctly depressed at frontoclypeal suture. Antenna with scape as long as pedicel. Pronotum with anterior margin broadly convex, middle carina obsolete, middle area of disk broad and explanate, lateral area gradually narrowing anteriorly and lateral margin nearly straight. Mesonotum with lateral carinae roundly curved. Veination of forewing complex, with dense crossveins, anterior margin slightly convex, apical margin broadly round, vein Sc with many branches, of which reaching anterior margin, R+M+Cu forking at base, apical cells distinctly long. Surface of forewings with sparse granules, area of clavus much denser. Hind tibiae with two lateral spines and seven apical spines, ventral surface groove lognitudinally.

**Figure 8.**
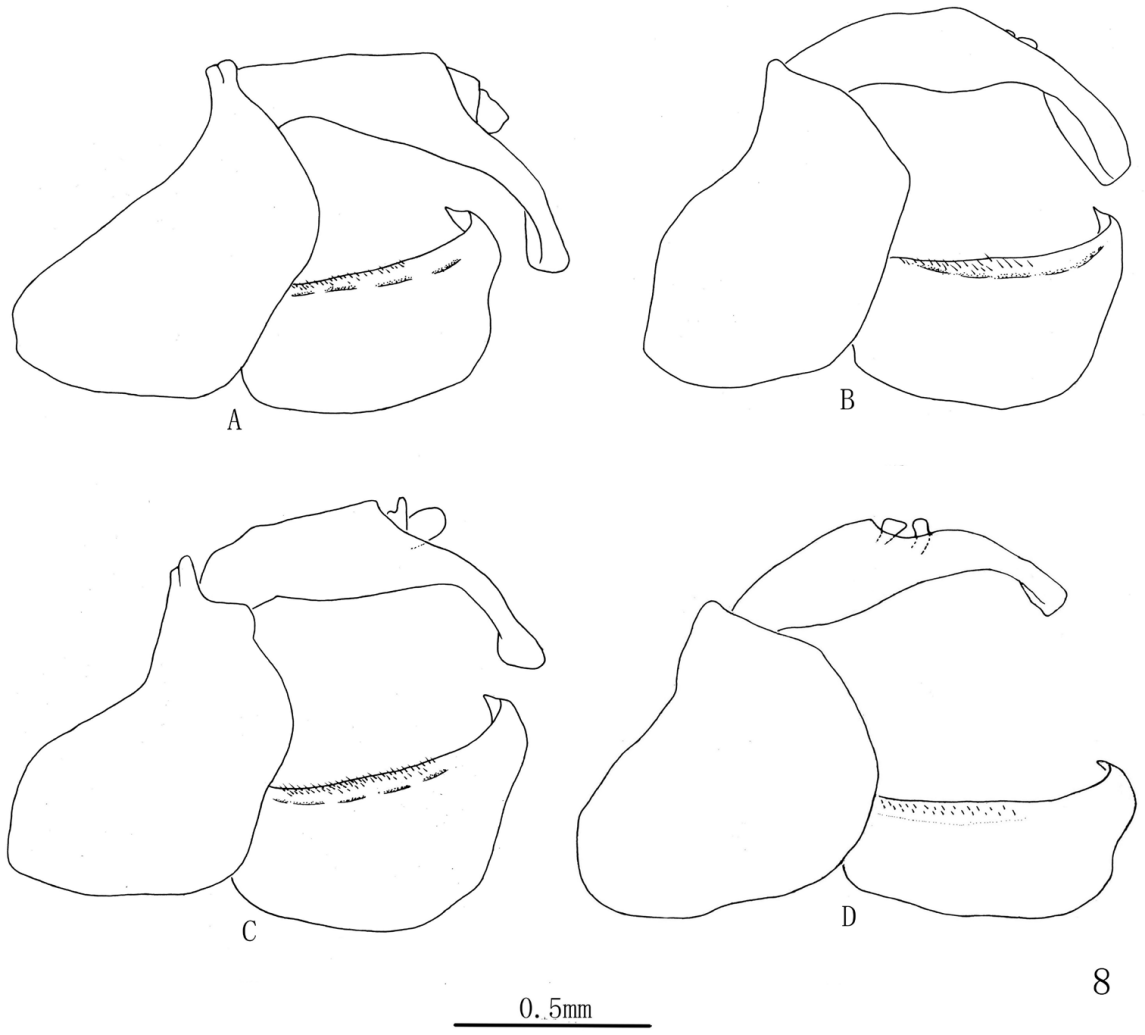
*Cerynia maria* (White, 1846) Variations of male pygofer, anal segment and genital style, lateral view. **A** (Guizhou, Guiyang; Guizhou, Luodian; Guizhou, Yanhe, Lijiaba; Guizhou, Maolan, Banzhai; Guizhou, Daozhen, Xiannvdong; Guizhou, Ziyun, Getuhe) **B** (Guangxi, Damingshan; Guizhou, Yanhe, Maojiacun; Guizhou, Xingyi, Muka) **C** (Guangxi, Longzhou; Guangxi, Daxing, Encheng; Guizhou, Liping, Taipingshan) **D**(Yunnan, Menglun).

**Figure 9.**
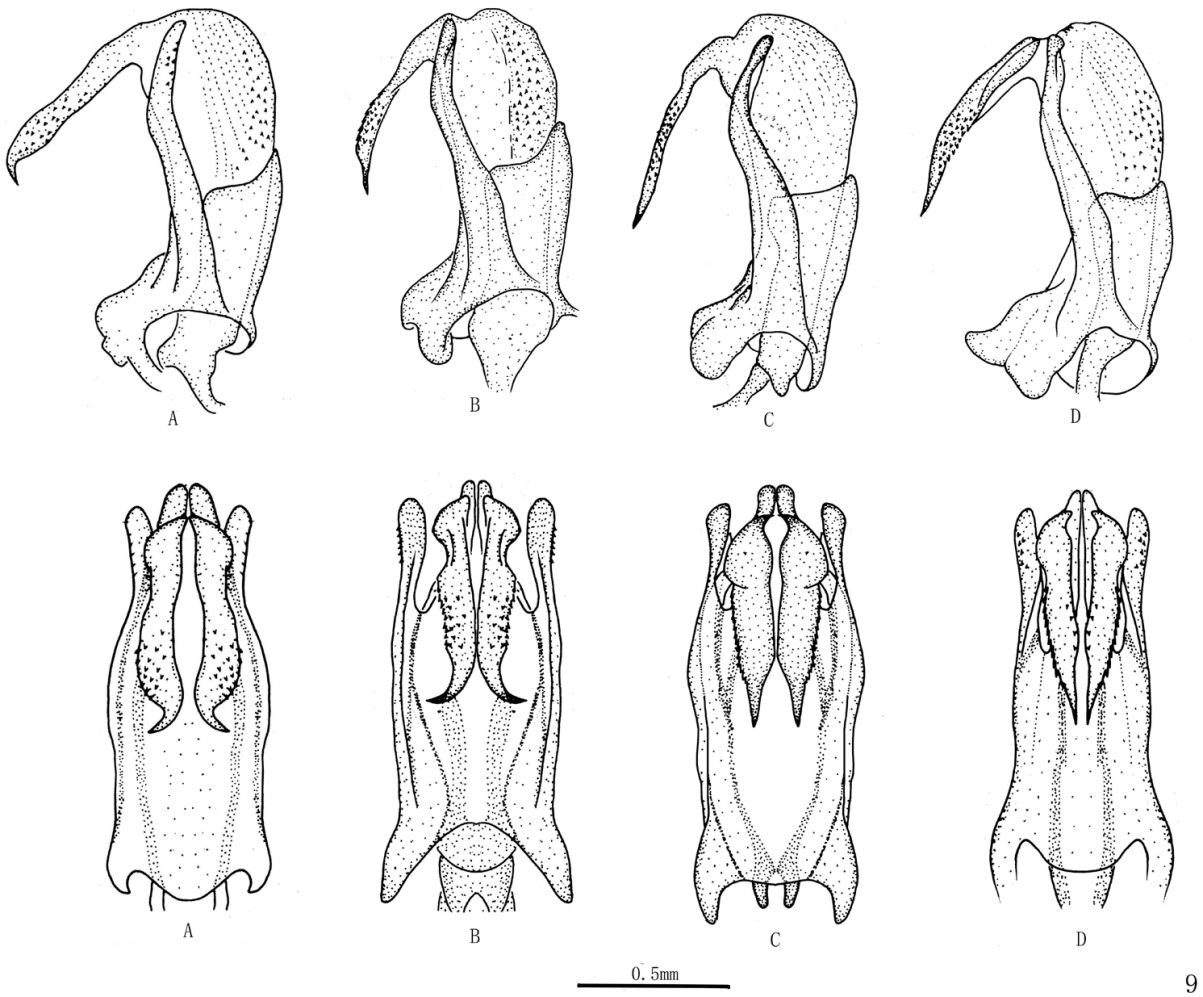
*Cerynia maria* (White, 1846) Variations of aedeagus, lateral and dorsal views. **A** (Guizhou, Guiyang; Guizhou, Luodian; Guizhou, Yanhe, Lijiaba; Guizhou, Maolan, Banzhai; Guizhou, Daozhen, Xiannvdong; Guizhou, Ziyun, Getuhe) **B** (Guangxi, Damingshan; Guizhou, Yanhe, Maojiacun; Guizhou, Xingyi, Muka) **C** (Guangxi, Longzhou; Guangxi, Daxing, Encheng; Guizhou, Liping, Taipingshan) **D** (Yunnan, Menglun).

***Male genitalia*.** Pygofer short, with lateral margin angle arced. Genital style subrectangular, with apex abruptly narrow and bending inward, apical margin sharp (Fig. 8). Aedeagus symmetrical; phallobase with base half tubular in lateral view, dorsolateral lobe slender, ventral lobe short; apex of theca with a pair of dorsal processes, of which with some small spines (Fig. 9).

Other external features see Table 1.

#### Distribution.

China (Fujian, Hainan, Yunnan, Guangxi, Hunan, Jiangxi, Guizhou); Vietnam; India; Burma.

#### Material examined.

A form: 2♂♂3♀♀, Guizhou, Guiyang, 4 July 1979, coll. Zizhong Li;18♂♂21♀♀, Guizhou, Guiyang, 5 May 1981, coll. Zizhog Li; 6♂♂10♀♀, Guizhou, Guiyang, 14 June 1983, coll. Zizhong Li; 2♂♂, Guizhou, Luodian, 5 June 1983, coll. Zizhong Li; 1♂2♀♀, Guizhou, Yanhe, Lijiaba, 7 June 2007, coll. Hansong Deng; 1♂, Guizhou, Maolan, Banzhai, 23 October 1998, coll. Xiangsheng Chen. 2♂♂, Guizhou, Daozhen, Xiannvdong, 28 May, 2004, coll. Qirong Liao; 7♂♂2♀♀, Guizhou, Ziyun, Getuhe, 24 June 2006, coll. Qiongzhang Song.

B form: 1♂, Guangxi, Damingshan, 15 May 2012, coll. Nanan Yang; 4♂♂4♀♀, Guizhou, Yanhe, Maojiacun, 9 June 2007, coll. Zhengguang Zhang; 2♂♂4♀♀, Guizhou, Xingyi, Muka, 17 May 1982, coll. Tandong Long.

C form: 1♂1♀, Guangxi, Longzhou, 30 May 1997, coll. Maofa Yang; 1♂2♀♀, Guangxi, Daxing, Encheng, 20 June 2012, coll. Weicheng Yang; 1♂9♀♀, Guizhou, Liping, Taipingshan, 16 July 2006, coll. Pei Zhang.

D form: 1♂1♀, Yunnan, Menglun, 28 July 2012, coll. Weicheng Yang.

#### Disscussion.

Colour polymorphism of *Cerynia maria* is investigated based on some materials from China, four colour forms are recongnized. The main differences in forewings with red markings or not, and apex of aedeagus dorsal processes bent or straight and number of its apical margin with spines.

Exploration of the molecular analysis (barcoding) about the species *Cerynia maria* is required but is not done so here because we did not succeed the genes about four colour forms of this species.

**Figure 10.**
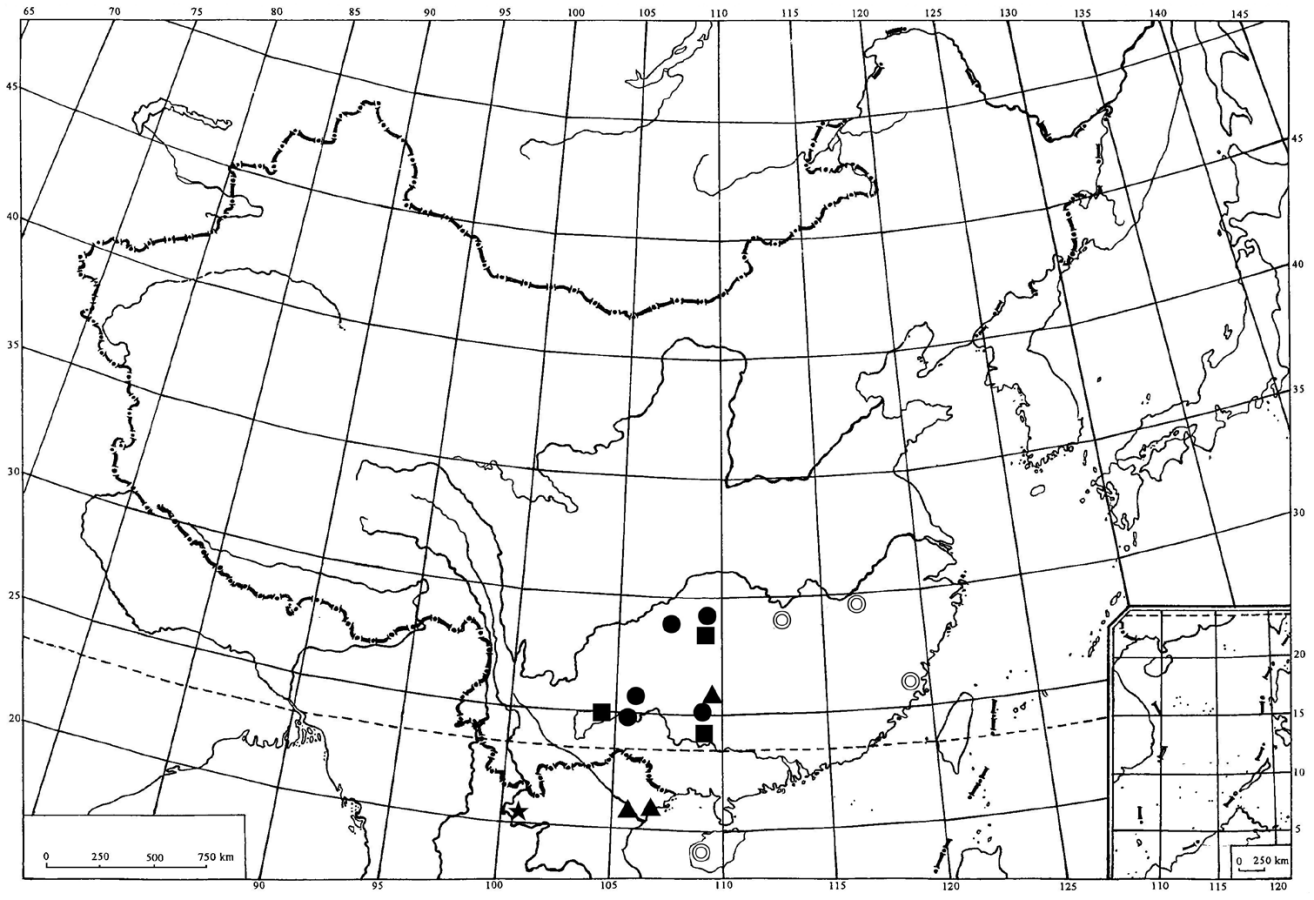
Geograghic distribution of *Cerynia maria* (White, 1846) from China. A form (●); B form (■); C form (▲); D form (★); unknown form (◎).

The geograghic distribution of *Cerynia maria* from China indicates that this species is mainly distributed in South China (Fig. 10). From our study materials from China show that three forms (A, B and C) distributed in Guizhou Province, B form and C form distributed in Guangxi Autonomous Region, and only D form found in Yunnan Province. However, it is highly likely that there are undiscovered forms in Fujian, Hainan, Hunan, Jiangxi and others Provinces from China.

**Table 1.** Differences variations of *Cerynia maria* (White, 1846).

	A	B	C	D
1. Forewings colour	Pink	White	White	Light green
2. Near base of forewings with red marking (number)	2	0	1	1
3. Ratio width of vertex from base to length in middle line	1.2	1.5	1.3	1.3
4. Ratio length of frons in middle line to maximum width	1.8	1.6	1.7	1.5
5. Ratio length of frons in middle line to length of clypeus	0.9	0.8	0.9	0.8
6. Ratio of mesonotum in middle line to length of pronotum	2.8	2.8	2.9	2.8
7. Ratio length of pronotum in middle line to length of vertex	0.9	0.9	1.0	0.8
8. Apex of aedeagus dorsal processes	Bent	Bent	Straight	Straight
9. Apical margin of aedeagus dorsal processes with spines	Many	Many	Only both sides	Only both sides

## Supplementary Material

XML Treatment for Cerynia lineola

XML Treatment for Cerynia maria
